# A systematic review of the opportunities and challenges of micro-credentials for multiple stakeholders: learners, employers, higher education institutions and government

**DOI:** 10.1186/s41239-023-00381-x

**Published:** 2023-02-28

**Authors:** Soovendran Varadarajan, Joyce Hwee Ling Koh, Ben Kei Daniel

**Affiliations:** Higher Education Development Centre, University of Otago, 65-75 Union Place West, P.O. Box 56, Dunedin, 9054 New Zealand

**Keywords:** Higher education, Micro-credentials, Student’s learning, Education system, Digital badges, Alternative credentials, Learning pedagogy

## Abstract

Micro-credentials are gaining traction as viable vehicles for rapid upskilling of the workforce in the twenty-first century and potential pathways for gaining employment for some students. The primary purpose of the current systematic review was to understand the current conceptions and discourses of micro-credentials in higher education and to identify the opportunities and challenges in adopting micro-credentials in higher education. The review also aimed to develop a need-driven micro-credentials framework that demonstrates the value of micro-credentials to stakeholders, i.e., learners, higher education institutions, employers, and government agencies. Key findings revealed that there are various stakeholders’ needs and expectations. The learner wants short, practical, and up-to-date courses for their chosen career path, education institutions emphasise accreditation for building trust, employers want clarity regarding the competencies gained through micro-credentials, and government bodies expect higher graduate employability with lower tuition fees. Key findings revealed that implementing micro-credentials can be disruptive in the higher education sector and present several challenges. However, these challenges are likely to be mitigated by increased collaboration among stakeholders. The review has revealed several outstanding research questions critical for the success of micro-credentials as significant pathways to supplement traditional degree programmes. The research presented in the article has implications for policy development to guide the implementation of micro-credentials in the higher education sector.

## Introduction

Scholarly discourses and research on micro-credentials have tremendously increased in recent years. While the concept of micro-credentials is not altogether newfound, interest has been rekindled and intensified since the aftermath of the pandemic COVID-19 (Brown et al., 2021). The potentials of micro-credentials gained the interests of increasingly diverse stakeholders, ranging from government officials, employers, learners, faculties, and most importantly higher education institutions across the globe. The recent attraction of micro-credentials can directly linked to declining student enrolment, especially after the COVID-19 pandemic, has made universities expand its reach to non-traditional student markets and international, geographically distant learners (McGreal & Olcott, 2022). This is done by tapping on online delivery modalities (Wheelahan & Moodie, 2021) one of which is through the awarding of micro-credentials.

The MicroHE Consortium (Uggeri & Barlassina, 2019) defined micro-credentials as a sub-unit of a credential that confers a minimum of 5 European Credit Transfer and Accumulation System (ECTS) and could accumulate into a more significant credential or be part of a portfolio. The New Zealand Qualification Authority (NZQA) bounds an upper and lower limit, defining micro-credentials as between 5 and 40 credits in size (Fisher & Leder, 2022).

In practice, micro-credentials have also, at times, been treated as synonymous with ‘digital badges,’ ‘open badges’, or ‘virtual badges’ that are digital tokens awarded upon completion of online learning modules (Clements et al., 2020). For example, digital badges can be used as certificates of assessed learning awarded by major MOOC platforms such as FutureLearn (UK), FUN (France), MiríadaX (Spain and IberoAmerica), EduOpen (Italy), and OpenupEd/ the European Association of Distance Teaching Universities (EADTU). The standards and assessment criteria of micro-credentials for these MOOC platforms were developed by European MOOC consortium through the development of the Common Microcredential Framework (CMF) which uses the European Qualification Framework (Fischer et al., 2022). Micro-credentials are seen as a way of meeting upskilling requirements for individuals looking to advance their career and to provide a skilled workforce for rapidly changing industries (Desmarchelier & Cary, 2022; Oliver, 2019). It is suggested that micro-credentials could improve access to higher education by decreasing the cost of enrolment (Wheelahan & Moodie, 2022).

While the positive impact of micro-credentials implementation has been upheld in many scholarly articles, there are numerous foreseeable challenges associated with the implementation of micro-credentialing in higher education. There is no global consensus on the definition and size of a micro-credentials as the term has been applied generically to individual courses and entire degree programmes (Wang et al., 2020). These variations persist, complicating the assessment and comparison of micro-credentials value for companies and learners (Cathrael Kazin & Clerkin, 2018). Micro-credentials tend to be accredited with digital badges, giving rise to perceptions of ‘badges’ eroding the status, credibility, and reputation of conventional qualifications awarded by the traditional academy (Mac Lochlainn et al., 2020). Opposing voices among conservatives who wish to preserve higher education as an ivory tower and support elite structures of higher education are also observed (Wheelahan & Moodie, 2022). Yet, in several countries, government bodies have keen interest in encouraging employers and learners to use micro-credentials as an alternative to the common education system (Ahmat et al., 2021). The varied definitions of micro-credentials, absence of accreditation frameworks, and the lack of organisational readiness appear to be challenges impeding the implementation of micro-credentials (Zhang & West, 2020).

While having micro-credentials alone will not be enough to fulfil any country’s future educational expectations, it nevertheless has the potential to expand and enhance the traditional university qualification systems through short and skill-based credit bearing programmes. Through micro-credentials, higher education pathways can potentially be created to support the continual acquisition of industry-relevant credentials even when people lack time or money to pursue a full degree programme (Carnevale et al., 2015). However, the potential of micro-credentials and the challenges of implementing them in higher education are still not well-understood (Zhang & West, 2020). This article aims to address this research gap through a systematic literature review of the extant of the published work. The review as guided by two research questions:

1. What is the conception of micro-credentials in higher education?
2. What are the opportunities and challenges associated with implementing micro-credentials in higher education?

## Methods and procedures

### Search strategy

A systematic search of the literature on micro-credentials was conducted between July 5, 2022, and August 30, 2022, using Scopus, ProQuest (ERIC), Web of Science (Core Collection) for SSCI-listed journals, and EBSCO Education Complete. The search procedure for this systematic review adhered to the PRISMA (Preferred Reporting Items for Systematic Reviews and Meta-Analyses) quality standards. The electronic search strategy for each database included a combination of the subject terms ‘micro-credentials,’ ‘digital badges,’ ‘open badge,’ ‘virtual badge,’ ‘micro-credentials’, and 'higher education.’ As an example, the search strategy for ProQuest (Eric) commenced with the significant subject terms ‘higher education’ and ‘micro-credentials.’ Subsequently, Boolean combinations of keywords were searched (without limits) for ‘micro-credit*’ OR ‘micro credent*’ OR ‘digital credent*’ OR ‘microcredent*’ AND ‘higher education’ OR ‘education’.

The search results from different databases were combined, and duplicates were eliminated, and inspected with the remaining records. Applying the selection criteria to the abstracts was the first step in the exclusion process. If abstracts were absent or offered inadequate information, papers were read in their entirety. Following this first abstract screening, the remaining publications were thoroughly examined with the following selection criteria to derive the final sample of records that satisfied all requirements:

- Publications covering the period 2015–2022. This period was chosen based on an initial database search, resulting in a higher volume of literature on the term ‘micro-credentials’ after 2015.
- The papers must be published in English or translated from the original language.
- Studies were required to describe the use of micro-credentials in the context of formal and non-formal education programmes, which included both formal academic education programmes in any field or profession such as engineering, medical, computer studies, business, social sciences, as well as programmes designed to provide professional development (i.e. continuing education) opportunities for working adults.
- Studies that provided insight into the possible benefits, design, implementation and evidence of this credentialing technique in the context of higher education programmes were included in the study.
- Studies about micro-credentials in the higher education were chosen. Studies about micro-credentials provided by third party learning platforms, learning academics, and other training organisations or centres were excluded.
- Empirical studies used quantitative, qualitative, or mixed methodologies or literature review papers.
- Quantifiable or thematic data has to be included in studies.
- Empirical studies must involve enrolled participants who participated in learning activities that included a micro-credentials as part of the lesson.

**Fig. 1:**
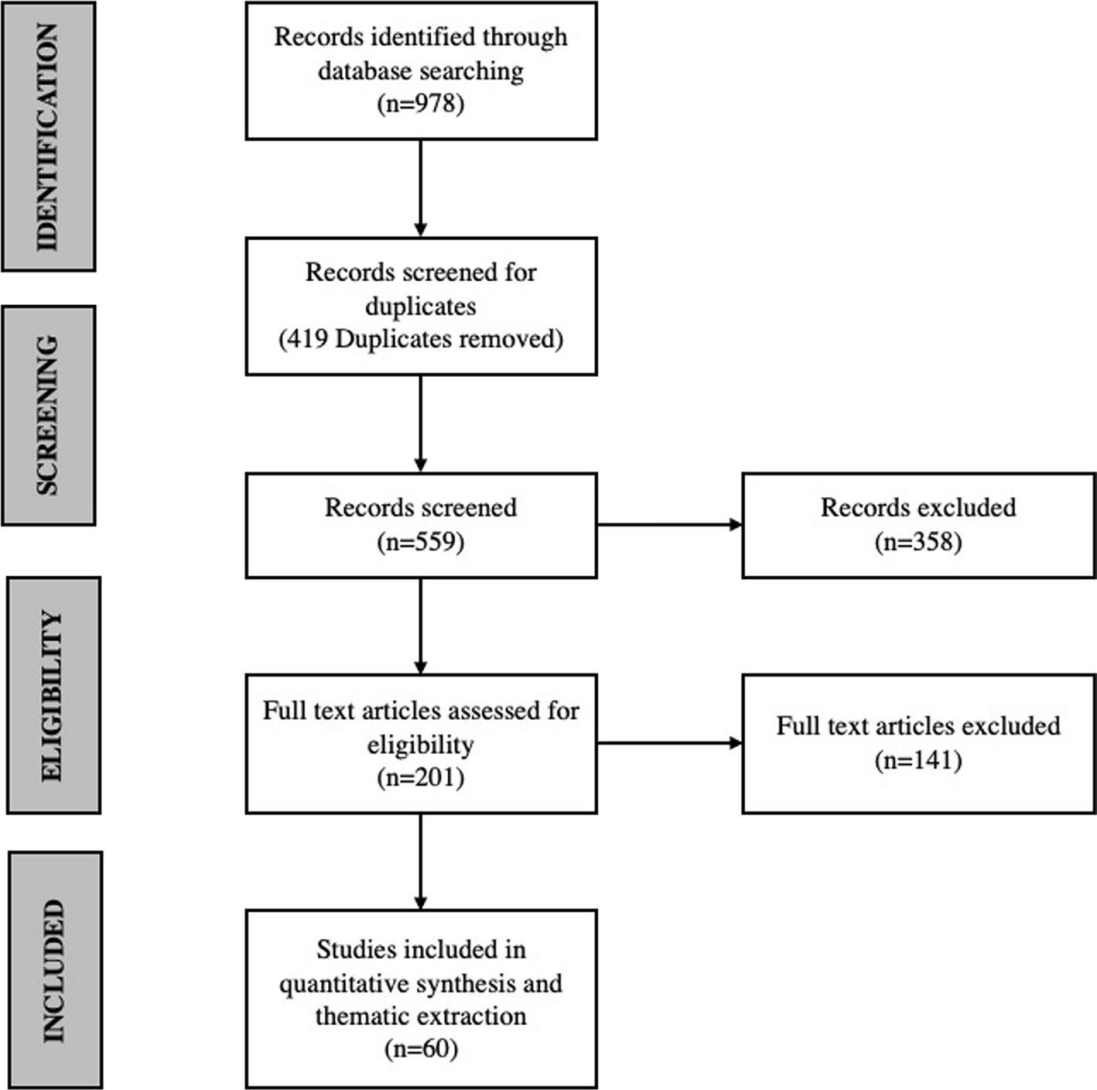
PRISMA (preferred reporting items for systematic reviews and meta-analysis)

Figure 1 depicts the database search and results in detail as a PRISMA flow diagram.

A listing of the papers selected is outlined in the coding table attached in Appendix 1.

### Data analysis

The articles were first coded for their general characteristics of year, study site, core theme, learning outcomes and definition/conceptualisation of micro-credentials to examine general research trends. Drawing upon this initial analysis, the research questions were examined through content analysis, as per Stemler (2000). This coding-and-counting method was selected and facilitated the inductive analysis of textual data into categories and the subsequent analysis of themes within and among categories. Texts relevant to the research questions were selected and categorised through a selective reduction procedure. The first author coded the articles and used the categories to formulate themes associated with the research questions. The two other authors reviewed and discussed the themes identified to ensure that there was consistency in the coding of the articles.

For the first research question, themes about the meaning and conception of micro-credentials in higher education were identified, and patterns among themes were discerned and discussed. The second part of the analysis involved coding for opportunities and challenges relating to implementing micro-credentials in higher education. Brown and Mhichil (2021) showed that students or learners, educational institutions, governments, and employers were the four key stakeholders of the micro-credentials ecosystem. It was found through coding the articles that stakeholder perspectives influenced what was considered opportunities and challenges. Therefore, stakeholder analysis (Brugha & Varvasovszky, 2000) was used to identify the critical player/s of micro-credentialing systems that each article focused on, the agendas of these stakeholders and how this influenced the opportunities and challenges reported in the articles.

### Summary of findings

**Fig. 2:**
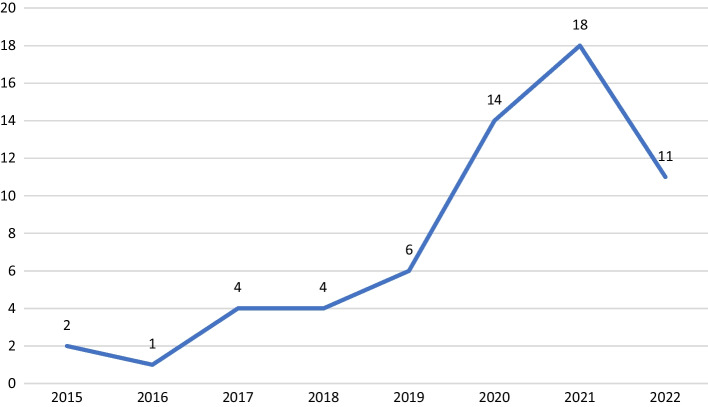
Publication trend

The review examined 60 selected studies published between 2015 and 2022. Of the total articles reviewed, 71% of the articles were published in the last 3 years (See Fig. 2). The trends in the research examining micro-credentialing after 2019 are consistent with recent reviews by Brown et al. (2021)

### Site of study

More than half of the 60 studies reviewed were from the United States of America (n = 27, 45%). This is followed by Australia (n = 14, 23%), Europe (n = 8, 13%) and Africa (n = 3, 5%). As a representation of the Asian region, three articles were reviewed from Malaysia and one from Istanbul. This is consistent with the trends in the keywords searched on “micro-credentials” or “microcredential” in the search engine Google (*Google *Trends, 2021).

### Stakeholder perspectives

Different stakeholders’ perspectives were represented in the discussion of the opportunities and challenges associated with micro-credentialing in higher education. All the articles reviewed mentioned one or more stakeholders in their articles. About 32% of the articles described higher education institutions’ perceptions on implementing micro-credentials. Learners’ perceptions follow this at 27%, and employers’ perceptions at 26%. Only 14% of the articles described governments’ perception of the implementation of micro-credentials.

### The choice of the term micro-credentials or microcredential

More than half of the publications (n = 42, 68%) used a hyphen to denote the term micro-credentials, while 32% (n = 19) of the publications used the term without a hyphen. This correspond with the research conducted by Lorenzo (2021), who explained that sometimes the term micro credential could be hyphenated. However, recent publications cited are mostly hyphenated.

### RQ1: the conception of micro-credentials

There is shared agreement of micro-credentials as shorter forms of a learning experience as compared to that of formal degree programmes—described by Oliver (2019) as a stackable certification of assessed learning that is additional, alternate, complementary to, or a formal component of a formal qualification that emphasises verified learning outcomes concerning traditional formal qualifications such as a bachelor’s or master’s degree (i.e., macro-credentials). Yet, there were variations in what constitutes a micro-credentials among the studies, depending on stakeholders and context.

#### Nomenclature

Sixty-eight per cent of the studies (n = 42) used the term micro-credentials with the inclusion of a hyphen, while 32% of the studies (n = 19) used the term micro-credentials without the hyphen. The nomenclature varied internationally, with the former more commonly adopted in North American studies. The rest of the studies used interchangeable terms, such as a digital badge, MOOC, micro-learning, etc. Twenty per cent (n = 12) of the studies considered micro-credentials as online qualifications, referring to them as digital badges and online certificates for massive open online courses (MOOCs). These studies were mainly describing micro-credentialing for learners who understood them as short courses that could be available online, completed at their own time, and student-centred, collaborative and interactive. Upon completion of micro-credentials courses, learners expect to be awarded a digital badge as a certification or acknowledgement of completion. These badges would be transferable across digital environments, including LinkedIn and social media. In the case of university-issued badges, they can also be attached to a student's academic transcript (Jones-Schenk, 2018). Six per cent of the studies (n = 4) and four per cent of the studies (n = 2) have used the term open badges and microlearning respectively and deem them as alternative credentials, nano-degrees, and micro-masters that may or may not necessarily be conducted online (Orr et al., 2020). The nomenclature adopted also varies by context. Government bodies often use the term micro-credentials to signify a qualification framework or an alternative education pathway but institutions of higher learning may treat micro-credentials as pedagogical tools, adopting the digital badges.

#### Size of programmes

The criteria used to demarcate the size of a micro-credentials varied among universities, industry, and government bodies. For example, concerning universities, Pickard et al. (2018) define a micro-credentials as any credential covering more than a single course but less than a full degree. However, more guidelines on credit value tend to be stipulated when considered from the industry or government perspectives. MicroHE, a European think-tank, view a micro-credentials as a “sub-unit of a credential or credentials that confer a minimum of 5 ECTS and could accumulate into a larger credential or be part of a portfolio” (Brown et al., 2021). This definition, which refers to study credits based on the European Credit Transfer System (ECTS), attempts to narrow the criteria for a micro-credentials in terms of course workload and hours of learning for the higher education sector.

#### Accreditation to macro-credentials

Twenty per cent of the studies (n = 12) briefly discussed the ways of accrediting micro-credentials to macro-credentials. For example, in New Zealand, micro-credentials is seen as a skill-oriented course that is currently not offered in any tertiary education system (Hartnett, 2021). On the other hand, micro-credentials may be offered as a precursor course (foundation level) for students looking to enter a diploma or a degree in higher education institutes in Malaysia (Ahmat et al., 2021). These examples show that the differences across regions and countries further complicate the implementation and recognition of micro-credentials.

### RQ2: what are the opportunities and challenges of embracing micro-credentials in higher education with respect to different stakeholders?

One of the most critical tasks during a new strategy is managing the interface between the many demands of an organisation’s different stakeholders concerning its strategic goals (Ackermann & Eden, 2011). There are four core stakeholders identified based on the systematic literature research conducted. The stakeholders are learners, higher education institutes, employers and government bodies. The following topics will elucidate the opportunities and challenges that were faced or will be faced by the stakeholders when embracing micro-credentials.

### Learners

Biesta (2015) has noted the recasting of students as ‘learners’ and the growing emphasis on the learner as the central figure within education. Learner-centredness is associated with the positioning of students as consumers of educational products (Standish, 2012). Students-as-consumers inhabit an educational ‘market’ where they may choose their preferred products and service providers, and the success of a transaction is measured through student satisfaction scores. These highly individualised consumers are imbued with the agency to act within their economic interests to increase their capital financing (Davies & Bansel, 2007). Institutions of higher education contemplate the ‘unbundling’ of their offerings to sell only those parts that the market desires (Swinnerton et al., 2020). Tables 1 and 2 list the opportunities and challenges faced or anticipated for learners due to implementing micro-credentials in a higher education institute.

**Table 1:** Summary view of the opportunities offered by micro-credentials for learners

Opportunities		N	Percentage (%)
LO1	Student centred learning / Increase Motivation	38	63
LO2	Increase flexibility for learning	36	60
LO3	Promote lifelong learning / Upskilling / Evidence of Skills	34	57
LO4	Increase employability	30	50
LO5	Develop 21st Century transversal skills	23	38
LO6	Increase access and pathways to formal education (cost)	13	22

**Table 2:** Summary view of the critical challenges offered by micro-credentials for learners

Challenges		N	Percentage (%)
LC1	Attainment of Knowledge	26	43
LC2	Credential specific to one particular job	20	33
LC3	No federal/standalone aid for micro-credentials / lack of funding	14	23

As per Table 1, micro-credentials is depicted as an alternative credential that increases employability (n = 30) through the opportunity to develop 21st-century skills such as digital literacy, communication, collaboration, critical thinking, problem-solving, decision making and creativity, which also improves employability (El Mawas & Muntean, 2018). From learners’ perspectives, it is perceived that micro-credentials provide them with opportunities for student-centred learning which increases their motivation to complete online learning courses (N = 38). This is followed by increased flexibility for learning (n = 36) and lifelong learning that provides evidence of skills attained (n = 34).

Among the critical challenges listed in Table 2, 43% of the literature reviewed discusses the narrower attainment of knowledge (n = 26) by learners, which gives rise to 33% of the articles discussing the limitation of the credential to one specific niche of the job scope (n = 20). Interestingly, only 23% of the studies (n = 14) reported that the lack of government aid for tuition fees was a challenge for adopting micro-credentials.

### Employer’s perspective

The labour market has changed in terms of employers’ expectations of new hires’ skills. Employers are looking for quick ways to train employees to boost productivity. At the same time, students want to learn a skill quickly so that they can return to work. As such, micro-credentialing is a potential mechanism to articulate the competencies that postsecondary institutions can provide students (Gauthier, 2020). Tables 3 and 4 illustrate the findings of opportunities and challenges of micro-credentials from employers’ perspectives respectively.

**Table 3:** Summary view of the opportunities offered by micro-credentials for employers

Opportunities		N	Percentage (%)
EO1	Fulfilling employers demands	45	75
EO2	Acknowledgment of skills	30	50
EO3	Promote sustainable development goals / CPD	25	42
EO4	Future of work and skills	23	38
EO5	Close skills gaps in response to changing nature of work	20	33

**Table 4:** Summary view of the challenges offered by micro-credentials for employers

Challenges		N	Percentage (%)
EC1	Consistency	48	80
EC2	Fraudulence / Authenticity due to variety	23	38
EC3	Lack of Formal Recognition	13	22

About 42% of the literature reviewed has mentioned that employers regard micro-credentialing as a pathway promoting sustainable continuous professional development (n = 42). Out of the 60 papers reviewed, about three-quarters have mentioned that employers believe that micro-credentials will be instrumental in fulfilling their demands for specific criteria and requirements (n = 45). This is followed by the acknowledgements of skills using digital badges at 50% (n = 30). Closing the skills gap in response to the changing nature of work is observed as one of the themes that emerged as opportunities from the employers' perspective, accounting for 33% of all the articles coded (n = 20).

Regarding challenges, almost 80% of the articles expressed employers’ concern about the consistency of micro-credentials (n = 42). Gauthier (2020) noted that micro-credentials must demonstrate completion and mastery of project-based education leading to competency in a given field or topic to avoid inconsistency in credential value. Micro-credentials integrity and the potential for fraudulent credentials were also expressed as challenges in 38% of the studies (n = 23). Only close to a quarter of the studies showed employers expressing concern for the lack of formal recognition for micro-credentials at 22% (n = 13).

### Educational institutions’ perspective

Several articles correlate the current upward trends in micro-credentials with the increasing neoliberal concepts in higher education institutions. Evidence of this is characterised by a shift in the privatisation and marketisation of public services (Brown et al., 2021; Reynoldson, 2022). There exists a tendency now to believe that every institution will participate in this developing market. McGreal and Olcott (2022) disagree, arguing that institutions must analyse and assess the environment to enter the market strategically, including an inventory of their institutional capability. Tables 5 and 6 below list the opportunities and challenges of implementing micro-credentials from educational institutions’ perspectives, respectively.

**Table 5:** Summary view of the opportunities offered by micro-credentials for the higher educational institutions

Opportunities		N	Percentage (%)
UO1	Support new models of pedagogy	46	77
UO2	Advancement in Technology	37	62
UO3	Develop a new 21st Century credential ecology	29	48
UO4	Test innovations and trigger changes	28	47
UO5	Promote major education system reform	21	35
UO6	Increase institution revenue and reputation	18	30
UO7	Reduce costs of education and training	11	18
UO8	Increase University Enrolment	9	15

**Table 6:** Summary view of the challenges in implementing micro-credentials in higher educational institutions

Challenges		N	Percentage (%)
UC1	Academic Support from the faculty / department / school / senior managers	55	92
UC2	Assessment of Credit / Credit Transfer / Value Defining / Accreditation of external qualification	14	23
UC3	Extensive review process by the faculty	9	15

Micro-credentials support new pedagogical models, as outlined in 77% of the papers reviewed (n = 46). This can be primarily due to the COVID-19 pandemic, which pushed learning into virtual spaces (Burrows et al., 2022; Maina et al., 2022; Olcott, 2022). Online learning paved a different version of pedagogical approaches and techniques from which micro-credentials aspects mainly benefit. The advancement of technology, such as Web2.0 Internet access, computer access, etc., makes the twenty-first century the best landscape to implement micro-credentials. This perception is echoed in 62% of the papers (n = 37). At most, 15% of the papers show institutions associating micro-credentials with increased revenue or enrolment (n = 9), whereas only 18% (n = 7) of the studies reflected institutions perceiving micro-credentials as advantageous for cost reduction (n = 11).

Majority of the articles reviewed expressed concerns related to the academic support of educators, faculty, students, and educational technologists in higher education environments (n = 55, 92%). This academic support can be inclusive of issues such as lack of understanding about micro-credentials among senior leaders and faculty members (Pickard et al., 2018), financial and funding allocation for university-wide implementation (Desmarchelier & Cary, 2022; Olcott, 2022), awareness of micro-credentials among teaching staffs (Ghasia et al., 2019). This is followed by an assessment of credits at 23% (n = 14). These assessment challenges can be inclusive of issues such as assessment on credit hours or notional time to complete a micro-credentials course, and valuation of credits from external sources for continuation into traditional formal credential (McGreal & Olcott, 2022). Fifteen per-cent of the articles reviewed express concern that the extensive review process by the faculty to implement micro-credentialing to be a challenge (n = 9).

### Governments’ perspective

The outbreak of the COVID-19 pandemic has mostly affected many functions of the governments. The micro-credentialing movement has provided opportunities for governments and higher education institutions to harness new digital learning models in partnership with industry. While the global micro-credentials landscape is currently fragmented across national borders, governments worldwide are expected to be increasingly aligning new credentialing developments with existing national qualification frameworks, bringing more clarity and coherence to the global micro-credentials landscape (Brown et al., 2021). The tables below reveal the opportunities and challenges in implementing micro-credentials as perceived by the governments (See Tables 7 and 8).

**Table 7:** Summary view of the opportunities and critical challenges offered by micro-credentials for government

Opportunities		N	Percentage (%)
GO1	Respond to changing learners’ demographics	24	40
GO2	Reflects Neo-liberal market forces	16	27
GO3	Respond to Pandemic—COVID-19	14	23
GO4	Globalisation & Growth	10	17
GO5	Increase equity for under-represented groups	8	13
GO6	Government Initiatives and Nationwide Policy	8	13

**Table 8:** Summary view of the critical challenges of micro-credentials implementation from governments’ perspective

Challenges		N	Percentage (%)
GC1	Confusions about the definitions and taxonomy	28	47
GC2	Funding and financing	19	32

Traditional tertiary education policy settings have mainly focused on younger learners in full-time study over lifelong learners. Forty per-cent of the articles (n = 24) raised matters related to micro-credentials being the tool to future-proof careers in response to the changing learners’ demographics shifting with the emergence of new industries, new ways of working, and developments in educational technology. This is followed by 27% (n = 16) of the articles discussing how governments can respond to neo-liberal market forces with micro-credentials. Most papers reviewed after 2020 related micro-credentials as a response to the COVID-19 pandemic (n = 14). While 13% (n = 8) of the articles mentioned government initiatives and nationwide policy implementation of micro-credentials, the federal government’s support in funding is still very low in countries such as Australia (Desmarchelier & Cary, 2022).

While there are many recent policy implementations towards alternative credentials, particularly on micro-credentials, by the government, 47% of the article's reviews expose the confusion about the actual definitions and taxonomy of the term micro-credentials (n = 28). The scope of the government body’s offer remains unknown, proof of their influence is limited, and government feedback on these new offerings has not been thoroughly recorded. This is followed by issues relating to funding and financing micro-credentials at public higher education institutions at 32% (n = 19). Kato et al. (2020) highlighted that governments tend not to extend higher education loans and grant programmes for traditional academic qualifications to alternative credentials. While their involvement in funding traditional courses is straightforward, some are reluctant to expose themselves to new and potentially risky investments in alternative credentials. Experimental or pilot initiatives in this area are also not forthcoming from government bodies.

## Discussion

**Fig. 3:**
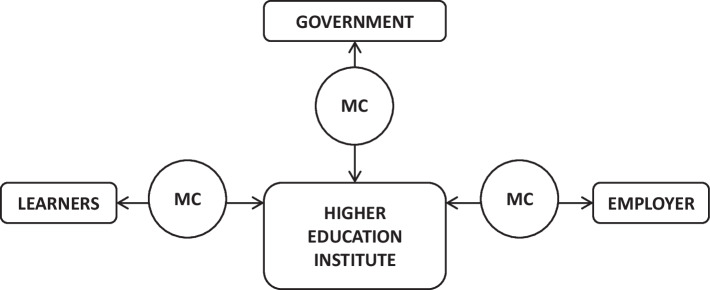
Higher education institution at the central position of the micro-credentials ecosystem

In an earlier review, Brown et al. (2021) show that the variation of nomenclature, credit size, and accreditation pathways used to define what constitutes a micro-credentials makes it confusing and bewildering to navigate the field. The findings of this review suggest that higher education institutions seeking to embark on micro-credentialing need to be aware of the critical stakeholders and their position within a larger ecosystem. Consistent with Brown and Mhichil (2021), who identified students or learners, educational institutions, governments and employers as the four key stakeholders of the micro-credentials ecosystem, our study findings suggest that higher education institutions can be visualised as the ones who are at the central position of the micro-credentials ecosystem (see Fig. 3).

From this perspective, we suggest three pathways for higher education institutions to implement micro-credentialing through more careful consideration of the opportunities and challenges of its ecosystem. There exists a prevalent assumption that all institutions will participate in this emerging market. While there appears to be demand from the industry, institutions must analyse and appraise the environment, including an inventory of their own institutional capabilities, in order to reset their priorities strategically to enter the market.

### Learners and higher education institutions—micro-credentials as a learning pathway

The first pathway is for higher education institutions to connect to learners through micro-credentialing. As reflected in the findings, from students’ perspectives, micro-credentials can be opportunities to acquire 21st-century skills in more student-centred ways. Learners value openness and transparency in participatory learning practices and peer-learning communities. This is in line with Devedzic and Jovanovic (2015) who argue that micro-credentials offer new ways of motivating learners and scaffolding the learning process. Higher education institutions, too, see micro-credentials as avenues for pedagogical innovation. Learners of the twenty-first century are both technically and technologically savvy, and the millennials’ state of mind is much changed from its previous generation, who placed high importance on a four-year college degree (Pace, 1990). This provides an opportunity for higher education institutions to revamp their strategy and policies about offering traditional courses that are typically longer in duration and higher in cost than micro-credentials.

Institutions need to develop comprehensive pathways from micro-credentials to macro-credential, which match the aspirations voiced by learners. Yet, there is concern about disruption in the higher education curricula because micro-credentials aimed at increasing employment might divert attention away from what successful education systems can accomplish (Buchanan et al., 2020). Ralston (2021) uses a Post digital Deweyan perspective to argue that micro-credentials are nothing more than an instance of ‘learning innovation theatre.’ Apart from the novelty component, Ralston (2021) asserts that higher education institutions sell their souls to business interests and market forces by unbundling degrees to boost revenues quickly. A renewed emphasis on future skills and vocational training has come at the price of holistic education. The fad is a betrayal of higher education’s greater purpose and a loss for students and professors who continue to view university education as more than vocational training. However, the COVID-19 pandemic has forced many institutions to change their brick-and-mortar course delivery and embrace online learning. This shows that the unbundling of higher education is already underway. Universities must become leaner, more efficient engines of innovation, firmly tied to local business due to enrolment issues, spiralling expenses, and the need to adapt to a changing global economy. If they do not, the new ‘upstarts,’ for-profit providers capable of offering certain services at a far lower price for the customer, will progressively displace them (McCowan, 2017). Financial barriers to undertaking micro-credentials do not comprise significant barriers for learners. Higher education institutions can offer micro-credentials in smaller units at lower costs than full-term tuition fees. Given the concern about the loss of revenue, the offering of cross-disciplinary or extra-curricular units as a requirement for the final accreditation of the full-stack course can be considered.

### Employers and higher education institutions—micro-credentials as an employment pathway

Forbes reported that in 2018, companies across the United States spent over $87.6 billion on corporate training and development (Pontefract, 2019). Employers can also use micro credential as digital credentials to shortlist candidates based on their required abilities for a position (Devedzic & Jovanovic, 2015). Employers are demanding people with skills and not merely paper qualifications (Kasriel, 2018). As shown in Table 4, employers are particularly interested in using micro-credentials. (Zhang & West, 2020) explained that many companies are exploring open micro-credentials as a form of professional microlearning. IBM’s expansive open micro-credentials initiative (see https://www-03.ibm.com/services/learning/) has issued open micro-credentials to their employees and others in their online training. IBM has reported that this program has resulted in greater employee engagement, more professional development participation, and higher social media brand impressions. Under a recent agreement with Northeastern University, many of these micro-credentials can now be converted to university credit for a master’s degree (Zhang & West, 2020). These developments show potential for a higher education-employer pathway through micro-credentials which can help employers to have sustainable development goals for their employees, support continuous professional development (CPD) and workplace training, and thereby help employers to reach a steady stream of workforce supply. However, as shown in the study results, a smaller proportion of papers reported employers seeing the legitimacy, trustworthiness, and consistency of micro-credentials offered by private vendors as a concern (Van Der Hijden (2019). Since higher education institutions have established academic rankings, systems and policies, these aspects of their brand image can be projected to micro-credentials, making them more authentic and credible to employers. These can also appeal to learners as they combine digital badges with their job application portfolios to add value during the recruiting process. Higher education institutions that can create consistency in micro-credentials structures linked to formal recognition can alleviate employers’ concerns. According to Selingo et al. (2018), American higher education is going through disruption, and the market for standard degrees is dwindling as the enthusiasm for micro-credentials continues. It has been predicted by the author of the Chronicle of Higher Education study “The Future of the Degree” that the standard bachelor’s degree will lose a lot of its value in the next decade (Selingo et al., 2018). In the US context, prior to the year before 2000, graduates are confirmed employment opportunities even before they complete their convocation ceremonies through campus interviews. However, this changed after the 2000s. The vast majority of the public lost confidence in a college certification as a torchbearer of social and economic attainments (Cochran-Smith, 2021). Observing the success of entrepreneurs like Bill Gates, Evan Williams, and Mark Zuckerberg—all of whom are college dropouts—the degree’s luster faded. Numerous prominent firms, including Google, Penguin Random House, and Apple, have eliminated the requirement for candidates to have a degree (Connley, 2018). As a result, the college diploma's currency as a guarantor of access to the professional workforce has dwindled. While higher education institutions do not provide micro-credentials to substitute traditional college and university degrees, they nevertheless can use micro-credentials to expand their market offerings by facilitating employers to use micro-credentials in conjunction with other forms of skill and knowledge certification. Higher education institutions can provide employers with the ability to trace and verify micro-credentials certification. They can also support employers with badges to acknowledge job hopefuls’ or even current workers’ talents that traditional grades and certificates frequently overlook. For instance, several efforts and projects have been developed around issuing badges for soft skills, such as critical thinking, skilled communication, collaboration, and resilience—qualities demanded by employers of job applicants (Devedzic & Jovanovic, 2015). By doing so, higher education institutions can potentially grow their student market through strategic partnerships with employers in talent management prior to, during, or after recruitment.

### Government and higher education institutions—micro-credentials as qualification framework

The study’s findings show that governments serve as regulatory bodies for the quality of educational offerings, directly or indirectly offering subsidies. With governments reducing funding for higher education, higher education has become more of a private commodity than a public asset as universities have increasingly had to seek funding (Ralston, 2021). On the other hand, governments are looking for a method to standardise, certify, recognise, and fund alternative credentials that can help them respond to emerging population demographics and societal problems at a lower cost.

There are two challenges majorly identified in the literature review. The first one is related to the awareness of micro-credentials among communities. As for the other challenge, micro-credentials is a robust approach to a century-old education method. Education institutions might not be able to support the fast-paced directions of these government agencies. They require momentum in order to shift the learning paradigm into micro-credentials mode. Thus, privatising micro-credentials offerings might be a solution, resulting in a bad reputation. Such as the sale of EDX by MIT (Saw, 2021). However, privatisation may assist in marketising micro-credentials to a higher height.

The three pathways outlined above outlined how higher education institutions can exploit micro-credentials by considering the perspectives of each key stakeholder in the micro-credentialing system. By doing so, higher education institutions can boost the likelihood that the micro-credentials system they set up can expand and contribute to the sustainability of the more enormous ecosystems, with them at the central position, as outlined in Fig. 3.

### Directions for future research

Considering micro-credentials are still in their early stages, a wide variety of research and perspectives can significantly contribute to its value and relevance. The present review of micro-credentials studies found a void in discussing institutions’ preparation for its deployment. While various perspectives of micro-credentials have been captured in the studies reviewed, more empirical studies are still needed to understand the level of preparedness of higher education institutions when implementing micro-credentials. For instance, more studies will be needed to address the richly diverse and different stakeholders’ expectations, how these interact at institutional and national levels. It will be imperative that specific guidelines that can help higher education institutions implement each of the three pathways outlined in Fig. 3 effectively can also be developed through Delphi studies with experts (Traxler et al., 2020). In future studies, the efficacy of the three pathways and how they can be implemented successfully can be empirically evaluated and validated.

Future more this research could be anchored to examine the effect of stacking micro-credentials on learners, specifically their perceptions and capacity to develop high-level competencies through micro-credentials (Cheng et al., 2020; Maina et al., 2022; McGreal & Olcott, 2022). Research could also investigate the relationship between the labour market needs and its demands in micro-credentials courses.

In addition the existing micro-credentials conceptions and frameworks, additional research could also be conducted on the roles and responsibilities of authorities responsible in determining the assessment criteria and qualification standard. These may provide additional value for the seamless implementation of micro-credentialing structure on a large scale. Further research could be conducted to provide the limitations of each stakeholder.

Finally, multiple frameworks exist on different delivery models of micro-credentials (Selvaratnam & Sankey, 2021b). An empirical analysis of micro-credentials as a credit bearing course could streamline the credit exemption process in higher education which would benefit the learners, the broader industry and government agencies.

## Limitations

There are various challenges and opportunities in implementing micro-credentials in a higher education institute. The purpose of this study was to understand these challenges and opportunities of a micro-credentials structure through the perspective of higher education institution. While there exist many adopters and implementers of micro-credentials such as MOOC platforms (e.g. Udacity, Coursera, EdX and etc.), learning centres or academies, and industries (IBM, Microsoft, Google and etc.), the limitation of this study is centred around higher education institutes. This is because there are more opportunities from higher education institution which acts as a linchpin among different stakeholders. Thus, the literatures reviewed represents a specific loci and the researcher understands that the findings and discussions from this study cannot be generally applied to all other providers or implementors of micro-credentials.

Another limitation of this study is that the results were not further broken down according to type of courses taken as micro-credentials such as theoretical or lab-based. While significant, the researchers wanted to focus on the holistic challenges and opportunities of higher education institutions as a whole in implementing micro-credentials.

## Conclusion

There is concern about how micro-credentials may decouple the traditional degree as institutions give in to the forces of a neoliberal learning economy (Ralston, 2021) take a similar stance, claiming that micro-credentials are ‘gig qualifications for a gig economy’ (p.8). However, this study demonstrates various possibilities amidst challenges for higher education institutions in the micro-credentials ecosystem. In an attempt to identify the trends in micro-credentials in the higher education sector, this paper has shown that the interaction among stakeholders is essential in shaping the future of micro-credentials. Higher education institutions serve as a crucial intersecting point for all other stakeholders. How they can effectively exploit to better support learners, employers, and government agencies can be further researched.

## Data Availability

All data generated or analysed during this study are included in this published article.

## Appendix 1

### Coding table

**Table:**

#	Author (Year)	Learners (L)	Employer (E)	Higher Education Institution (U)	Government Bodies (G)
JA01	Devedzic and Jovanovic (2015)	[LO1], [LO2], [LO3], [LO5]	[EC1], [EC2], [EO1], [EO3], [EO5]	[UC1], [UC3], [UO1], [UO2], [UO3]	[GC1], [GO1], [GO4]
JA02	Law (2015)	[LC1], [LC2], [LO1], [LO2], [LO5], [LO6]	[EC1], [EO3], [EO4]	[UC1], [UO1], [UO2], [UO3]	
JA03	Loeckx (2016)	[LC3], [LO1], [LO2]	[EC1], [EO1], [EO2]	[UC1], [UO1], [UO2], [UO3], [UO4], [UO5], [UO6]	[GC1], [GC2], [GO5]
JA04	Gauthier (2020)	[LC1], [LO2], [LO3], [LO4]	[EC1], [EC2], [EO1], [EO2], [EO3]	[UC1], [UO2], [UO4]	[GC1], [GO1], [GO4]
JA05	Dyjur and Lindstrom (2017)	[LC3], [LO1], [LO2]	[EC1], [EC2], [EC3], [EO1]	[UC1], [UO1], [UO2], [UO6], [UO7], [UO8]	[GC1], [GC2], [GO1]
JA06	Foshay and Hale (2017)	[LO1], [LO2], [LO3], [LO5]	[EC1], [EC2], [EO1], [EO3], [EO5]	[UC1], [UC3], [UO1], [UO2], [UO3]	[GC1], [GO1]
JA07	LaMagna (2017)	[LC1], [LC2], [LO1], [LO2], [LO5], [LO6]	[EC1], [EO3], [EO4]	[UC1], [UO1], [UO2], [UO3]	
JA08	Reeves et al. (2017)	[LC3], [LO1], [LO2]	[EC1], [EO1], [EO2]	[UC1], [UO1], [UO2], [UO3], [UO4], [UO5], [UO6]	[GC1], [GC2], [GO4], [GO5]
JA09	Carey and Stefaniak (2018)	[LO2], [LO3], [LO4]	[EC1], [EC2], [EO1]	[UC1], [UO2], [UO4]	[GO1]
JA10	Cheng et al. (2018)	[LC1], [LO1], [LO2]	[EC1]	[UC1], [UO1], [UO2]	[GO1]
JA11	Daellenbach (2018)	[LO1], [LO2], [LO4], [LO6]	[EC1], [EO1], [EO4]	[UC1], [UC2], [UO1], [UO2], [UO4], [UO5]	
JA12	Jones-Schenk (2018)	[LC1], [LO1], [LO3], [LO5], [LO6]	[EC1], [EO1], [EO2], [EO4], [EO5]	[UC1], [UO1], [UO2]	[GO4]
JA13	Hoanca and Craig (2019)	[LC1], [LC2], [LO1], [LO2], [LO5], [LO6]	[EC1], [EO3], [EO4]	[UC1], [UO1], [UO2], [UO3]	
JA14	Ruddy and Ponte (2019)	[LC2], [LO1], [LO3], [LO4], [LO5]	[EO2], [EO4], [EO5]	[UC1], [UO1], [UO2], [UO3], [UO4]	
JA15	Young et al. (2019)	[LC1], [LO2], [LO3], [LO4]	[EC1], [EC2], [EO1], [EO2], [EO3]	[UC1], [UO1], [UO2], [UO4]	[GC1], [GO1], [GO4]
JA16	Ghasia et al. (2019)	[LC3], [LO1], [LO2]	[EC1], [EC2], [EC3], [EO1]	[UC1], [UO1], [UO2], [UO6], [UO7], [UO8]	[GC1], [GC2], [GO1], [GO4]
JA17	Zucker and Hicks (2019)	[LO1], [LO3], [LO5]	[EC1], [EC2], [EO1], [EO3], [EO5]	[UC1], [UC3], [UO1], [UO2], [UO3]	[GC1], [GO1]
JA18	Clements et al. (2020)	[LC1], [LC2], [LO1], [LO5], [LO6]	[EO3], [EO4]	[UC1], [UO1], [UO3]	
JA19	Gan (2020)	[LC3], [LO1]	[EC1], [EO1], [EO2]	[UC1], [UO1], [UO3], [UO4], [UO5], [UO6]	[GC1], [GC2], [GO2], [GO5]
JA20	Goodenough et al. (2020)	[LC1], [LO2], [LO3], [LO4]	[EC1], [EC2], [EO1], [EO2], [EO3]	[UC1], [UO4]	[GC1], [GO1]
JA21	Hunsaker and West (2020)	[LC3], [LO1], [LO2]	[EC1], [EC2], [EC3], [EO1]	[UC1], [UO1], [UO2], [UO6], [UO7], [UO8]	[GC1], [GC2], [GO1], [GO2]
JA22	Hunt et al. (2020)	[LO1], [LO2], [LO4], [LO6]	[EO1], [EO4]	[UC1], [UC2], [UO1], [UO4], [UO5]	[GO4]
JA23	Krause (2022)	[LC1], [LO1], [LO3], [LO5], [LO6]	[EC1], [EO1], [EO2], [EO4], [EO5]	[UC1], [UO1], [UO2]	
JA24	Newby and Cheng (2020)	[LC1], [LC2], [LO1], [LO2], [LO5], [LO6]	[EC1], [EO3], [EO4]	[UC1], [UO1], [UO2], [UO3]	[GO2]
JA25	Peacock et al. (2020)	[LC2], [LO1], [LO2], [LO3], [LO4], [LO5]	[EO2], [EO4], [EO5]	[UC1], [UO1], [UO2], [UO3], [UO4]	[GO4]
JA26	Pence (2020)	[LC3], [LO1]	[EC1], [EO1], [EO2]	[UC1], [UO1], [UO2], [UO3], [UO4], [UO5], [UO6]	[GC1], [GC2], [GO5]
JA27	Randall and West (2020)	[LC1], [LO3], [LO4]	[EC1], [EC2], [EO1], [EO2], [EO3]	[UC1], [UO2], [UO4]	[GC1], [GO1], [GO2]
JA28	Risquez and Cassidy (2020)	[LO3], [LO5]	[EC1], [EC2], [EC3], [EO3], [EO4], [EO5]	[UC1], [UC3], [UO1], [UO4], [UO7]	[GC1], [GO1], [GO4], [GO6]
JA29	Spencer and Bussi (2020)	[LC3], [LO1]	[EO1], [EO2]	[UC1], [UO1], [UO2], [UO3], [UO4], [UO5], [UO6]	[GC1], [GC2], [GO5]
JA30	Spencer (2020)	[LC1], [LO3], [LO4]	[EC2], [EO1], [EO2], [EO3]	[UC1], [UO2], [UO4]	[GC1], [GO1], [GO2]
JA31	West et al. (2020)	[LC1], [LC2], [LO1], [LO2], [LO5], [LO6]	[EC1], [EO3], [EO4]	[UC1], [UO1], [UO2], [UO3]	
JA32	Zhang and West (2020)	[LC1], [LC2], [LO1], [LO2], [LO4], [LO6]	[EC1], [EO1], [EO2], [EO3], [EO4]	[UC1], [UC2], [UO1], [UO2]	[GC1], [GO1]
JA33	Brown et al. (2021)	[LC3], [LO1]	[EC1], [EO1], [EO2]	[UC1], [UO1], [UO2], [UO3], [UO4], [UO5], [UO6]	[GC1], [GC2], [GO5]
JA34	Clausen (2021)	[LC1], [LO3], [LO4]	[EC1], [EC2], [EO1], [EO2], [EO3]	[UC1], [UO4]	[GC1], [GO1], [GO2]
JA35	Cook (2021)	[LC1], [LO1]	[EC1], [EC2], [EO5]	[UC1], [UO1]	[GC1]
JA36	Gish-Lieberman et al. (2021)	[LO1], [LO2], [LO3]	[EC1]	[UC1], [UO1]	
JA37	Gonzalez and Villaire (2021)	[LC2], [LO2], [LO3], [LO5], [LO6]	[EC1], [EO1], [EO2], [EO4], [EO5]	[UC1], [UC2], [UO3]	[GO2]
JA38	Hartnett (2021)	[LC1], [LC2], [LO1], [LO2], [LO3], [LO5]	[EC1], [EC3], [EO1], [EO4], [EO5]	[UC1], [UO1], [UO5]	[GO2], [GO6]
JA39	Klotzbach-Russell et al. (2021)	[LC3], [LO1]	[EC1], [EO1], [EO2]	[UC1], [UO1], [UO3], [UO4], [UO5], [UO6]	[GC1], [GC2], [GO5]
JA40	Perkins and Pryor (2021)	[LC1], [LO3], [LO4]	[EC1], [EC2], [EO1], [EO2], [EO3]	[UC1], [UO1], [UO4]	[GC1], [GO1]
JA41	Selvaratnam and Sankey (2021b)	[LC2], [LO1], [LO2], [LO3], [LO4], [LO5]	[EC1], [EC3], [EO1], [EO2], [EO3]	[UC1], [UC2], [UO1], [UO3], [UO4], [UO5], [UO8]	[GC1], [GO1], [GO2], [GO3]
JA42	Wheelahan and Moodie (2022)	[LC1], [LC2], [LO2], [LO3], [LO4], [LO5]	[EC1], [EC2], [EC3], [EO1], [EO5]	[UC1], [UC2], [UO1], [UO3], [UO4], [UO6], [UO7], [UO8]	[GC2], [GO1], [GO3]
JA43	Wheelahan and Moodie (2021)	[LC1], [LC2], [LO1], [LO4], [LO5]	[EC1], [EC2], [EO1]	[UC1], [UC2], [UO1], [UO3], [UO5], [UO6]	[GC2], [GO2], [GO3]
JA44	Woods and Woods (2021)	[LC3], [LO3], [LO4]	[EO1], [EO2], [EO3], [EO4], [EO5]	[UC3], [UO1], [UO2], [UO6], [UO8]	[GO2], [GO3], [GO6]
JA45	Ralston (2021)	[LC1], [LC2], [LO2], [LO3], [LO4]	[EC1], [EC2], [EC3], [EO1], [EO2]	[UC1], [UC2], [UO2]	[GC2], [GO3]
JA46	Selvaratnam and Sankey (2021a)	[LC2], [LO1], [LO2], [LO3], [LO4], [LO5]	[EC1], [EC3], [EO1], [EO2], [EO3]	[UC1], [UC2], [UO1], [UO2], [UO3], [UO4], [UO5], [UO8]	[GC1], [GO1], [GO2], [GO3]
JA47	Ahmat et al. (2021)	[LC1], [LC2], [LO2], [LO3], [LO4], [LO5]	[EC1], [EC2], [EC3], [EO1], [EO5]	[UC1], [UC2], [UO1], [UO3], [UO4], [UO6], [UO7], [UO8]	[GC2], [GO1], [GO2], [GO3]
JA48	Boud and Jorre de St Jorre (2021)	[LC1], [LC2], [LO1], [LO4], [LO5]	[EC1], [EC2], [EO1]	[UC1], [UC2], [UO1], [UO3], [UO5], [UO6]	[GC2], [GO3]
JA49	Martinez-Marroquin and Male (2021)	[LC3], [LO3], [LO4]	[EO1], [EO2], [EO3], [EO4], [EO5]	[UC3], [UO1], [UO2], [UO6], [UO8]	[GO2], [GO3], [GO6]
JA50	Chukowry et al. (2021)	[LC1], [LC2], [LO2], [LO3], [LO4]	[EC1], [EC2], [EC3], [EO1], [EO2]	[UC1], [UC2], [UO1], [UO2]	[GC2], [GO3]
JA51	Yilmaz et al. (2022)	[LO1], [LO2], [LO3]	[EO2], [EO5]	[UC1], [UC3], [UO1], [UO2], [UO3], [UO4]	
JA52	Burrows et al. (2022)	[LO1], [LO2], [LO3]		[UC1]	[GO3]
JA53	Desmarchelier and Cary (2022)	[LC1], [LC2], [LC3], [LO2], [LO4]	[EC1], [EO3], [EO4], [EO5]	[UC2], [UO1], [UO3], [UO5], [UO6], [UO7]	[GC2], [GO1], [GO2], [GO3], [GO6]
JA54	Kumar Jeya et al. (2022)	[LO2], [LO3], [LO4]	[EO1]	[UC1], [UO1], [UO2], [UO7]	[GC1], [GO3]
JA55	Maina et al. (2022)	[LO3], [LO4]	[EC1], [EO1], [EO2], [EO4]	[UO1], [UO4], [UO5]	[GO5]
JA56	Reynoldson (2022)	[LO1], [LO2], [LO4]	[EC1], [EC3], [EO1], [EO4]	[UC1], [UO3], [UO5]	[GC1], [GC2], [GO1], [GO2], [GO6]
JA57	McGreal and Olcott (2022)	[LO3], [LO4], [LO6]	[EC1], [EO1], [EO2], [EO3], [EO5]	[UC1], [UC3], [UO2], [UO4], [UO5], [UO7]	[GO3], [GO4], [GO6]
JA58	Felton et al. (2022)	[LO3]	[EO1], [EO4], [EO5]	[UC1], [UO2], [UO4], [UO5], [UO7]	[GO6]
JA59	Miller and Jorre de St Jorre (2022)	[LO3], [LO4], [LO5]	[EC1], [EC2], [EO1], [EO2], [EO4], [EO5]	[UO5]	
JA60	Olcott (2022)	[LC3], [LO1], [LO2], [LO4]	[EC1], [EC3], [EO1], [EO3]	[UC1], [UC2], [UC3], [UO3], [UO5], [UO6], [UO7]	[GC1], [GC2], [GO1]

### Legend of coding table

**Table:**

Employer’s Perspective		
Challenges	EC1	Consistency
	EC2	Fraudulence / Authenticity due to variety
	EC3	Lack of Formal Recognition
Opportunities	EO1	Employers Demands
	EO2	Acknowledgment of Skills
	EO3	Promote Sustainable Development Goals / CPD
	EO4	Future of Work & Skills
	EO5	Close skills gaps in response to changing nature of work
Governments’ Perspective		
Challenges	GC1	Confusions about the definitions and taxonomy
	GC2	Funding and Financing
Opportunities	GO1	Respond to changing demographics
	GO2	Reflects Neo-liberal market forces
	GO3	Respond to Pandemic—COVID-19
	GO4	Globalisation & Growth
	GO5	Increase equity for under-represented groups
	GO6	Government Initiatives and Nationwide Policy
Learners’ Perspective		
Challenges	LC1	Attainment of Knowledge
	LC2	Credential specific to one particular job
	LC3	Not federal aid standalone for micro-credentials / lack of funding
Opportunities	LO1	Student centred learning / Increase Motivation
	LO2	Increase flexibility for learning
	LO3	Promote lifelong learning / Upskilling / Evidence of Skills
	LO4	Increase employability
	LO5	Develop 21st Century transversal skills
	LO6	Increase access and pathways to formal education (cost)
Higher Education Institutes’ Perspective		
Challenges	UC1	Implementation—Academic, lack of funding
	UC2	Assessment of Credit / Credit Transfer / Value Defining / Accreditation of external qualification
	UC3	Extensive review process by the faculty
Opportunities	UO1	Support new models of pedagogy
	UO2	Advancement in Technology
	UO3	Develop a new 21st Century credential ecology
	UO4	Test innovations and trigger changes
	UO5	Promote major education system reform
	UO6	Increase institution revenue and reputation
	UO7	Reduce costs of education and training
	UO8	Increase University Enrolment

## References

[CR1] Ackermann F, Eden C (2011). Strategic management of stakeholders: Theory and practice. Long Range Planning.

[CR2] Ahmat NHC, Bashir MAA, Razali AR, Kasolang S (2021). Micro-credentials in higher education institutions: Challenges and opportunities. Asian Journal of University Education.

[CR3] Biesta GJ (2015). Beyond learning: Democratic education for a human future.

[CR4] Boud D, de St Jorre T (2021). The move to micro-credentials exposes the deficiencies of existing credentials. Journal of Teaching and Learning for Graduate Employability.

[CR5] Brown, M., & Mhichil, M. N. G. (2021). Unboxing micro-credentials: An inside, upside and downside View. https://www.dcu.ie/sites/default/files/inline-files/unboxing-micro-credentials-2021.pdf

[CR6] Brown M, Mhichil MNG, Beirne E, Mac Lochlainn C (2021). The global micro-credential landscape: Charting a new credential ecology for lifelong learning. Journal of Learning for Development.

[CR7] Brugha R, Varvasovszky Z (2000). Stakeholder analysis: A review. Health Policy and Planning.

[CR8] Buchanan, J., Allais, S., Anderson, M., Calvo, R. A., Peter, S., & Pietsch, T. (2020). The futures of work: what education can and can’t do. *United Nations Educational, Scientific and Cultural Organisation, Paris*. https://unesdoc.unesco.org/ark:/48223/pf0000374435

[CR9] Burrows AC, Borowczak M, Mugayitoglu B (2022). Computer science beyond coding: partnering to create teacher cybersecurity microcredentials. Education Sciences.

[CR10] Carey KL, Stefaniak JE (2018). An exploration of the utility of digital badging in higher education settings [Article]. Educational Technology Research and Development.

[CR11] Carnevale, A. P., Smith, N., Melton, M., & Price, E. (2015). Learning While Earning: The New Normal. *Georgetown University Center on Education and the Workforce*. http://hdl.voced.edu.au/10707/396609

[CR12] Cathrael Kazin, J., & Clerkin, K. M. (2018). *The potential and limitations of microcredentials*. http://supportsystem.livehelpnow.net/resources/23351/Potential%20and%20Limitations%20of%20Microcredentials%20FINAL_SEPT%202018.pdf

[CR13] Cheng Z, Richardson JC, Newby TJ (2020). Using digital badges as goal-setting facilitators: A multiple case study [Article]. Journal of Computing in Higher Education.

[CR14] Cheng Z, Sunnie Lee W, Newby TJ (2018). Goal setting and open digital badges in higher education. TechTrends.

[CR15] Chukowry V, Nanuck G, Sungkur RK (2021). The future of continuous learning–Digital badge and microcredential system using blockchain. Global Transitions Proceedings.

[CR16] Clausen JM (2021). Learning to fly: Development and design of a micro-credentialing system for an educator preparation program in the absence of a required educational technology course. TechTrends.

[CR17] Clements K, West RE, Hunsaker E (2020). Getting started with open badges and open microcredentials. International Review of Research in Open and Distributed Learning.

[CR18] Cochran-Smith M (2021). Rethinking teacher education: The trouble with accountability. Oxford Review of Education.

[CR94] Connley, C. (2018). Google, Apple and 12 other companies that no longer require employees to have a college degree. CNBC, 8 October. https://www.cnbc.com/2018/08/16/15-companies-that-no-longer-require-employees-to-have-a-college-degree.html. Accessed 20 May 2020

[CR19] Cook E (2021). Practice-based engineering: Mathematical competencies and micro-credentials. International Journal of Research in Undergraduate Mathematics Education.

[CR20] Daellenbach K (2018). On carrot cake and marketing education: A perspective on balancing skills for employability. Australasian Marketing Journal (AMJ).

[CR21] Davies B, Bansel P (2007). Neoliberalism and education. International Journal of Qualitative Studies in Education.

[CR22] Desmarchelier R, Cary LJ (2022). Toward just and equitable micro-credentials: an Australian perspective: Revista de Universidad y Sociedad del Conocimiento. International Journal of Educational Technology in Higher Education.

[CR23] Devedzic V, Jovanovic J (2015). Developing open badges: A comprehensive approach. Etr&d-Educational Technology Research and Development.

[CR24] Dyjur P, Lindstrom G (2017). Perceptions and uses of digital badges for professional learning development in higher education [Article]. TechTrends.

[CR25] Felton SD, Whitehouse G, Motley C, Jaeger D, Timur A (2022). How I stopped fearing micro-credentials and began to love digital badging—a pilot project [Article]. Industry and Higher Education.

[CR26] Fisher RM, Leder H (2022). An assessment of micro-credentials in New Zealand vocational education. International Journal of Training Research.

[CR27] Fischer, T., Oppl, S., & Stabauer, M. (2022). Micro-Credential Development: Tools, Methods and Concepts Supporting the European Approach. 17th International Conference on Wirtschaftsinformatik, Nuremberg, Germany, February 2022. https://aisel.aisnet.org/wi2022/digital_education/digital_education/1

[CR28] Foshay WR, Hale J (2017). Application of principles of performance-based assessment to corporate certifications. TechTrends.

[CR29] Gan CY (2020). Full cycle of micro-credentials trial: A case study from a Malaysian private university [Article]. Journal of Advanced Research in Dynamical and Control Systems.

[CR30] Gauthier T (2020). The value of microcredentials: The employer's perspective. The Journal of Competency-Based Education.

[CR31] Ghasia, M., Machumu, H., & Smet, E. (2019). *Micro-credentials in higher education institutions: An exploratory study of its place in Tanzania*. Open Campus, The University of the West Indies, West Indies. Retrieved December 26, 2021 from https://www.learntechlib.org/p/209746/

[CR32] Gish-Lieberman JJ, Tawfik A, Gatewood J (2021). Micro-credentials and badges in education: A historical overview. TechTrends.

[CR33] Gonzalez DP, Villaire M (2021). Advancing professional development in health literacy: The health literacy specialist certificate program and the health literacy solutions center. American Journal of Health Education.

[CR34] Goodenough B, Watts J, Bartlett S (2020). Making sense of self-reported practice impacts after online dementia education: The example of Bedtime to Breakfast and beyond [Article]. Brain Impairment.

[CR35] *Google Trends*. (2021). https://trends.google.com/trends/explore?date=today%205-y&q=microcredential,microcredential,micro%20credential

[CR36] Hartnett M (2021). How and why are digital badges being used in higher education in New Zealand? [Article]. Australasian Journal of Educational Technology.

[CR37] Hoanca, B., & Craig, B. (2019). Building a K-16-industry partnership to train IT professionals [Article]. *Journal of Information Systems Education*, *30*(4), 232–241. https://www.scopus.com/inward/record.uri?eid=2-s2.0-85081176142&partnerID=40&md5=7df4aa13bca7896c263828acb9e458b7

[CR38] Hunsaker E, West RE (2020). Designing computational thinking and coding badges for early childhood educators. TechTrends.

[CR39] Hunt T, Carter R, Yang S, Zhang L, Williams M (2020). Navigating the use of microcredentials. Journal of Special Education Technology.

[CR40] Jones-Schenk J (2018). Alternative credentials for workforce development. Journal of Continuing Education in Nursing.

[CR93] Kasriel, S. (2018). The future of work won’t be about college degrees, it will be about job skills. CNBC, 8 November. https://www.cnbc.com/2018/10/31/the-future-of-work-wont-be-about-degrees-it-will-be-about-skills.html. Accessed 20 Aug 2021.

[CR41] Kato S, Galán-Muros V, Weko T (2020). The emergence of alternative credentials. OECD Education Working Papers.

[CR42] Klotzbach-Russell C, Rowley EM, Starry R (2021). Librarians in the LaunchPad: Building partnerships for entrepreneurial information literacy. Journal of Business & Finance Librarianship.

[CR43] Krause K-LD (2022). Vectors of change in higher education curricula. Journal of Curriculum Studies.

[CR44] Kumar Jeya A, Richard Rachel J, Osman S, Lowrence K (2022). Micro-credentials in leveraging emergency remote teaching: the relationship between novice users’ insights and identity in Malaysia: Revista de Universidad y Sociedad del Conocimiento. International Journal of Educational Technology in Higher Education.

[CR45] LaMagna M (2017). Placing digital badges and micro-credentials in context [Article]. Journal of Electronic Resources Librarianship.

[CR46] Law P (2015). Digital badging at The Open University: Recognition for informal learning. Open Learning.

[CR47] Loeckx J (2016). Blurring boundaries in education: Context and impact of MOOCs. International Review of Research in Open and Distributed Learning.

[CR48] Lorenzo, G. (2021). *The Rapidly Changing Global Landscape of Microcredentials*. Retrieved 23 from https://evolllution.com/programming/credentials/the-rapidly-changing-global-landscape-of-microcredentials/

[CR49] Mac Lochlainn C, Nic Giolla Mhichíl M, Beirne E, Brown M (2020). The soul behind the screen: Understanding cultural enrichment as a motivation of informal MOOC learning. Distance Education.

[CR50] Maina MF, Guàrdia Ortiz L, Mancini F, Martinez Melo M (2022). A micro-credentialing methodology for improved recognition of HE employability skills: Revista de Universidad y Sociedad del Conocimiento. International Journal of Educational Technology in Higher Education.

[CR51] Martinez-Marroquin, E., & Male, S. (2021). Micro-credentials for recognition of workplace learning: Provocation. *Journal of Teaching and Learning for Graduate Employability*, *12*(1), 52–57. https://ojs.deakin.edu.au/index.php/jtlge/article/view/1513

[CR52] El Mawas, N., & Muntean, C. (2018). *Supporting lifelong learning through development of 21 st century skills* 10th International Conference on Education and New Learning Technologies, https://hal.archives-ouvertes.fr/hal-02250150

[CR91] McCowan, T. (2017). Higher education unbundling and the end of the university as we know it. *Oxford Review of Education, 43*(6), 733–748. 10.1080/03054985.2017.1343712

[CR53] McGreal R, Olcott D (2022). A strategic reset: Micro-credentials for higher education leaders. Smart Learning Environments.

[CR54] Miller KK, de St J, Jorre T (2022). Digital micro-credentials in environmental science: An employer perspective on valued evidence of skills [Article]. Teaching in Higher Education.

[CR55] Newby TJ, Cheng Z (2020). Instructional digital badges: Effective learning tools [Article]. Educational Technology Research and Development.

[CR56] Olcott D (2022). Micro-credentials: A catalyst for strategic reset and change in U.S. higher education [Article]. American Journal of Distance Education.

[CR92] Oliver, B. (2019). Making micro-credentials work for learners, employers and providers. Retrieved from http://hdl.voced.edu.au/10707/515939.

[CR57] Orr D, Pupinis M, Kirdulyte G (2020). Towards a European approach to micro-credentials: A study of practices and commonalities in offering micro-credentials in European higher education. Publications Office of the European Union.

[CR58] Pace, C. R. (1990). The Undergraduates: A Report of Their Activities and Progress in College in the 1980’s. https://files.eric.ed.gov/fulltext/ED375701.pdf

[CR59] Peacock R, Grevatt H, Dworak E, Marsh L, Doty S (2020). Developing and evaluating an asynchronous online library microcredential: A case study. Reference Services Review.

[CR60] Pence HE (2020). How should chemistry educators respond to the next generation of technology change?. Education Sciences.

[CR61] Perkins J, Pryor M (2021). Digital badges: Pinning down employer challenges [Article]. Journal of Teaching and Learning for Graduate Employability.

[CR62] Pickard, L., Shah, D., & De Simone, J. (2018). *Mapping microcredentials across MOOC platforms* 2018 Learning With MOOCS (LWMOOCS), Madrid, Spain. https://ieeexplore.ieee.org/stamp/stamp.jsp?tp=&arnumber=8534617

[CR63] Pontefract, D. (2019). *The Wasted Dollars Of Corporate Training Programs*. https://www.forbes.com/sites/danpontefract/2019/09/15/the-wasted-dollars-of-corporate-training-programs/?sh=1f4f720b71f9

[CR64] Ralston SJ (2021). Higher education’s microcredentialing craze: a Postdigital-Deweyan critique. Postdigital Science and Education.

[CR65] Randall DL, West RE (2020). Who cares about open badges? An examination of principals’ perceptions of the usefulness of teacher open badges in the United States [Article]. Open Learning.

[CR66] Reeves TD, Tawfik AA, Msilu F, Simsek I (2017). What’s in it for me? Incentives, learning, and completion in massive open online courses. Journal of Research on Technology in Education.

[CR67] Reynoldson M (2022). Marketing micro-credentials: An analysis of actors, voices and messages in educational innovation discourse [Article]. Innovations in Education and Teaching International.

[CR68] Risquez A, Cassidy D (2020). Badge of honour? An exploration of the use of digital badges to support a partnership approach to faculty development [Article]. Australasian Journal of Educational Technology.

[CR69] Ruddy C, Ponte F (2019). Preparing students for university studies and beyond: A micro-credential trial that delivers academic integrity awareness. Journal of the Australian Library and Information Association.

[CR70] Saw, J. (2021). *Harvard and MIT to Sell edX for $800 Million*. https://www.harvardmagazine.com/2021/06/sale-of-edx

[CR71] Selingo, J. J., Clark, C., & Noone, D. (2018). *The future(s) of public higher education: How state universities can survive-and thrive-in a new era*. https://www2.deloitte.com/content/dam/insights/us/articles/4726_future-of-higher-education/DI_Future-of-public-higher-ed.pdf

[CR72] Selvaratnam R, Sankey M (2021). The state of micro-credentials implementation and practice in Australasian higher education. Open Praxis.

[CR73] Selvaratnam RM, Sankey MD (2021). An integrative literature review of the implementation of microcredentials in higher education: Implications for practice in Australasia [Article]. Journal of Teaching and Learning for Graduate Employability.

[CR74] Spencer A, Bussi A (2020). The university language centre as an open-badge issuer: New directions in ESP assessment and accreditation. Language Learning in Higher Education.

[CR75] Spencer M (2020). Micro-credentialing as making and doing STS. Learning Communities-International Journal of Learning in Social Contexts.

[CR76] Standish A (2012). The false promise of global learning: Why education needs boundaries.

[CR77] Stemler S (2000). An overview of content analysis. Practical Assessment, Research, and Evaluation.

[CR78] Swinnerton B, Coop T, Ivancheva M, Czerniewicz L, Morris NP, Swartz R, Walji S, Cliff A, Dohn NB, Jandric P, Ryberg T, de Laat M (2020). The Unbundled University Researching emerging models in an unequal landscape. Mobility, data and learner agency in networked learning.

[CR79] Traxler, J., Scott, H., Smith, M., & Hayes, S. (2020). Learning through the crisis: Helping decision-makers around the world use digital technology to combat the educational challenges produced by the current COVID-19 pandemic. https://edtechhub.org/

[CR80] Van Der Hijden, P. (2019). Digitization of Credentials: Quality of Shorter-Term Educational Experiences. *Council for Higher Education Accreditation*. https://files.eric.ed.gov/fulltext/ED597931.pdf (CHEA/CIQG Publication Series)

[CR81] Uggeri, M., & Barlassina, L. (2019). Challenges and opportunities of micro-credentials in Europe. http://hdl.voced.edu.au/10707/590350

[CR82] Wang C, Kantor CM, Mitchell JT, Bacastow TS, Guo H, Goodchild MF, Annoni A (2020). Digital earth education. Manual of digital earth.

[CR83] West RE, Tawfik AA, Gishbaugher JJ, Gatewood J (2020). Guardrails to constructing learning: The potential of open microcredentials to support inquiry-based learning. TechTrends.

[CR84] Wheelahan L, Moodie G (2021). Analysing micro-credentials in higher education: A Bernsteinian analysis [Article]. Journal of Curriculum Studies.

[CR85] Wheelahan L, Moodie G (2022). Gig qualifications for the gig economy: Micro-credentials and the ‘hungry mile’. Higher Education.

[CR86] Woods K, Woods JA (2021). Less is more: Exploring the value of micro-credentials within a graduate program [Article]. Journal of Continuing Higher Education.

[CR87] Yilmaz Y, Papanagnou D, Fornari A, Chan TM (2022). The learning loop: Conceptualizing just-in-time faculty development [Article]. AEM Education and Training.

[CR88] Young D, West RE, Nylin TA (2019). Value of open microcredentials to earners and issuers: a case study of national instruments open badges. International Review of Research in Open and Distributed Learning.

[CR89] Zhang J, West RE (2020). Designing microlearning instruction for professional development through a competency based approach. TechTrends.

[CR90] Zucker L, Hicks T (2019). Alternative assessments, unintended consequences: The promise and peril of digital badges. Transformations.

